# A New Apparatus for Maintaining Anæsthesia without a Face-Piece, and with the Mouth Open

**Published:** 1893-10

**Authors:** Thomas Fillebrown

**Affiliations:** Boston, Mass.


### A NEW APPARATUS FOR MAINTAINING ANÆSTHESIA WITHOUT A FACE-PIECE, AND WITH THE MOUTH OPEN.[1]

BY DR. THOMAS FILLEBROWN, BOSTON, MASS.

THE necessity of repeatedly reanæsthetizing the patient has always been a great and serious hinderance to the progress and success of surgical operations within and about the mouth and throat. My own experience in operating upon the palate and lips led me to consider the possibility of maintaining the narcosis without interfering with the operation. I am pleased to announce the accomplishment of this object.

Several anæsthetists have used etherized air in inducing and continuing anæsthesia. Clover used chloroform in this way many years ago, and later applied ether the same way. Messrs. Codman & Shurtleff, of Boston, produced an apparatus for this purpose some years ago.

The working of all of the inhaling instruments depended upon the force of the inspiration of the patient to draw the air through the ether, and all except Codman & Shurtleff's provided for the patient to expire back into the instrument.

About two years ago Dr. Horace Packard used a compressible bulb to force the air through the ether, and thus made a very great

---

[1] Read before the World's Columbian Dental Congress, Chicago, August, 1893.

improvement over the old method; but this still allowed the respiration to pass back into the instrument. The observations of the workings of this inhaler led me to the conception of an apparatus to accomplish the much-desired object of anæsthetizing without sponge, towel, or face-piece.

My apparatus consists of a bellows, connected by rubber tubing with the long tube of a twelve-ounce wash-bottle, with a stop-cock intervening to regulate the flow of air. From the bottle extends a half-inch rubber tube to the patient. The bottle is filled one-third full of ether. The bellows is inflated and the stop-cock opened so as to allow the air to bubble up freely through the ether, and to become saturated with ether vapor. The etherized air is then discharged through the second tube a few inches from the patient's face.

This application of ether will maintain complete anæsthesia for any length of time, and not interfere in the least with any operation in or about the mouth; nor will the surplus vapor discharged into the air sensibly affect either the operator or the assistants.

I have maintained perfect anæsthesia for half an hour in one case to one and one-half hour in another case, without intermitting the operation at all on account of the anæsthesia. This method is not wasteful of ether, as less than one-half pound continued the narcosis the hour and a half; but the waste I should consider of no account as compared with the great advantage gained.

I have made a still further modification of the instrument, and adapted it to the induction of initial anæsthesia, and its maintenance with face-piece for use in general surgery.

All ether inhalers now in use allow all the exhalations to be forced back into the instrument; consequently the patient breathes over and over again the same atmosphere and becomes more or less asphyxiated, instead of truly anæsthetized. Such a plan seems to me unscientific, uncleanly, and unhealthy, in every way undesirable.

To avoid this condition, I have attached to the discharge-tube of the apparatus above described a double-valve face-piece, such as is used for the administration of nitrous oxide, and a double-end gas bag for a reservoir. By this means the patient is insured an abundance of pure anæsthetic atmosphere for each inhalation.

At the suggestion of Dr. T. M. Dillingham, I first filled the bag with pure air, and by slowly injecting the etherized air, narcosis is induced with absolute freedom from any of the disagreeable symptoms usually experienced when etherization is accomplished.

I have since found that the same object is better accomplished by raising the tube in the bottle until it is entirely free from the ether, filling the bag, and commencing the inhalation. In this air there will be but a very little ether. Then gradually slide the tube down into the bottle, and as it approaches the ether the strength is increased; after one-fourth of a minute the patient can breathe the full strength.

If the mouth is to be operated on, when anæsthesia is complete disconnect the bag and face-piece and proceed as before described.

